# Systematic mapping of existing tools to appraise methodological strengths and limitations of qualitative research: first stage in the development of the CAMELOT tool

**DOI:** 10.1186/s12874-019-0728-6

**Published:** 2019-06-04

**Authors:** Heather Menzies Munthe-Kaas, Claire Glenton, Andrew Booth, Jane Noyes, Simon Lewin

**Affiliations:** 1 Norwegian Institute of Public Health, Oslo, Norway; 2 School of Health and Related Research (ScHARR), University of Sheffield, Sheffield, UK; 3 School of Social Sciences, Bangor University, Bangor, UK; 4 Health Systems Research Unit, South African Medical Research Council, Cape Town, South Africa

**Keywords:** Methodological limitations, Qualitative research, Qualitative evidence synthesis, Systematic mapping, Framework synthesis

## Abstract

**Background:**

Qualitative evidence synthesis is increasingly used alongside reviews of effectiveness to inform guidelines and other decisions. To support this use, the GRADE-CERQual approach was developed to assess and communicate the confidence we have in findings from reviews of qualitative research. One component of this approach requires an appraisal of the methodological limitations of studies contributing data to a review finding. Diverse critical appraisal tools for qualitative research are currently being used. However, it is unclear which tool is most appropriate for informing a GRADE-CERQual assessment of confidence.

**Methodology:**

We searched for tools that were explicitly intended for critically appraising the methodological quality of qualitative research. We searched the reference lists of existing methodological reviews for critical appraisal tools, and also conducted a systematic search in June 2016 for tools published in health science and social science databases. Two reviewers screened identified titles and abstracts, and then screened the full text of potentially relevant articles. One reviewer extracted data from each article and a second reviewer checked the extraction. We used a best-fit framework synthesis approach to code checklist criteria from each identified tool and to organise these into themes.

**Results:**

We identified 102 critical appraisal tools: 71 tools had previously been included in methodological reviews, and 31 tools were identified from our systematic search. Almost half of the tools were published after 2010. Few authors described how their tool was developed, or why a new tool was needed. After coding all criteria, we developed a framework that included 22 themes. None of the tools included all 22 themes. Some themes were included in up to 95 of the tools.

**Conclusion:**

It is problematic that researchers continue to develop new tools without adequately examining the many tools that already exist. Furthermore, the plethora of tools, old and new, indicates a lack of consensus regarding the best tool to use, and an absence of empirical evidence about the most important criteria for assessing the methodological limitations of qualitative research, including in the context of use with GRADE-CERQual.

**Electronic supplementary material:**

The online version of this article (10.1186/s12874-019-0728-6) contains supplementary material, which is available to authorized users.

## Background

Qualitative evidence syntheses (also called systematic reviews of qualitative evidence) are becoming increasingly common and are used for diverse purposes [1]. One such purpose is their use, alongside reviews of effectiveness, to inform guidelines and other decisions, with the first Cochrane qualitative evidence synthesis published in 2013 [2]. However, there are challenges in using qualitative synthesis findings to inform decision making because methods to assess how much confidence to place in these findings are poorly developed [3]. The ‘Confidence in the Evidence from Reviews of Qualitative research’ (GRADE-CERQual) approach aims to transparently and systematically assess how much confidence to place in individual findings from qualitative evidence syntheses [3]. Confidence here is defined as “an assessment of the extent to which the review finding is a reasonable representation of the phenomenon of interest” ([3] p.5). GRADE-CERQual draws on the conceptual approach used by the GRADE tool for assessing certainty in evidence from systematic reviews of effectiveness [4]. However, GRADE- CERQual is designed specifically for findings from qualitative evidence syntheses and is informed by the principles and methods of qualitative research [3, 5].

The GRADE-CERQual approach bases its assessment of confidence on four components: the methodological limitations of the individual studies contributing to a review finding; the adequacy of data supporting a review finding; the coherence of each review finding; and the relevance of a review finding [5]. In order to assess the methodological limitations of the studies contributing data to a review finding, a critical appraisal tool is necessary. Critical appraisal tools “provide analytical evaluations of the quality of the study, in particular the methods applied to minimise biases in a research project” [6]. Debate continues over whether or not one should critically appraisal qualitative research [7–15]. Arguments against using criteria to appraise qualitative research have centred on the idea that “research paradigms in the qualitative tradition are philosophically based on relativism, which is fundamentally at odds with the purpose of criteria to help establish ‘truth’” [16]. The starting point in this paper, however, is that it is both possible and desirable to establish a set of criteria for critically appraising the methodological strengths and limitations of qualitative research. End users of findings from primary qualitative research and from syntheses of qualitative research often make judgments regarding the quality of the research they are reading, and this is often done in an ad hoc manner [3]. Within a decision making context, such as formulating clinical guideline recommendations, the implicit nature of such judgements limits the ability of other users to understand or critique these judgements. A set of criteria to appraise methodological limitations allows such judgements to be conducted, and presented, in a more systematic and transparent manner. We understand and accept that these judgements are likely to differ between end users – explicit criteria help to make these differences more transparent.

The terms “qualitative research” and “qualitative evidence synthesis” refer to an ever-growing multitude of research and synthesis methods [17–20]. Thus far, the GRADE-CERQual approach has mostly been applied to syntheses producing a primarily descriptive rather than theoretical type of finding [5]. Consequently, it is primarily this descriptive standpoint from which the analysis presented in the current paper is conducted. The authors acknowledge, however, the potential need for different criteria when appraising the methodological strengths and limitations of different types of primary qualitative research. While accepting that there is probably no universal set of critical appraisal criteria for qualitative research, we maintain that some general principles of good practice by which qualitative research should be conducted do exist. We hope that our work in this area, and the work of others, will help us to develop a better understanding of this important area.

In health science environments, there is now widespread acceptance of the use of tools to critically appraise individual studies, and as Hannes and Macaitis have observed, “it becomes more important to shift the academic debate from whether or not to make an appraisal to what criteria to use” [21]. This shift is paramount because a plethora of critical appraisal tools and checklists [22–24] exists and yet there is little, if any, agreement on the best approach for assessing the methodological limitations of qualitative studies [25]. To the best of our knowledge, few tools have been designed for appraising qualitative studies in the context of qualitative synthesis [26, 27]. Furthermore, there is a paucity of tools designed to critically appraise qualitative research to inform a practical decision or recommendation, as opposed to critical appraisal as an academic exercise by researchers or students.

In the absence of consensus, the Cochrane Qualitative & Implementation Methods Group (QIMG) provide a set of criteria that can be used to select an appraisal tool, noting that review authors can potentially apply critical appraisal tools specific to the methods used in the studies being assessed, and that the chosen critical appraisal tool should focus on methodological strengths and limitations (and not reporting standards) [11]. A recent review of qualitative evidence syntheses found that the majority of identified syntheses (92%; 133/145) reported appraising the quality of included studies. However, a wide range of tools were used (30 different tools) and some reviews reported using multiple critical appraisal tools [28]. So far, authors of Cochrane qualitative evidence syntheses have adopted different approaches, including adapting existing appraisal tools and using tools that are familiar to the review team.

**Fig. 1:**
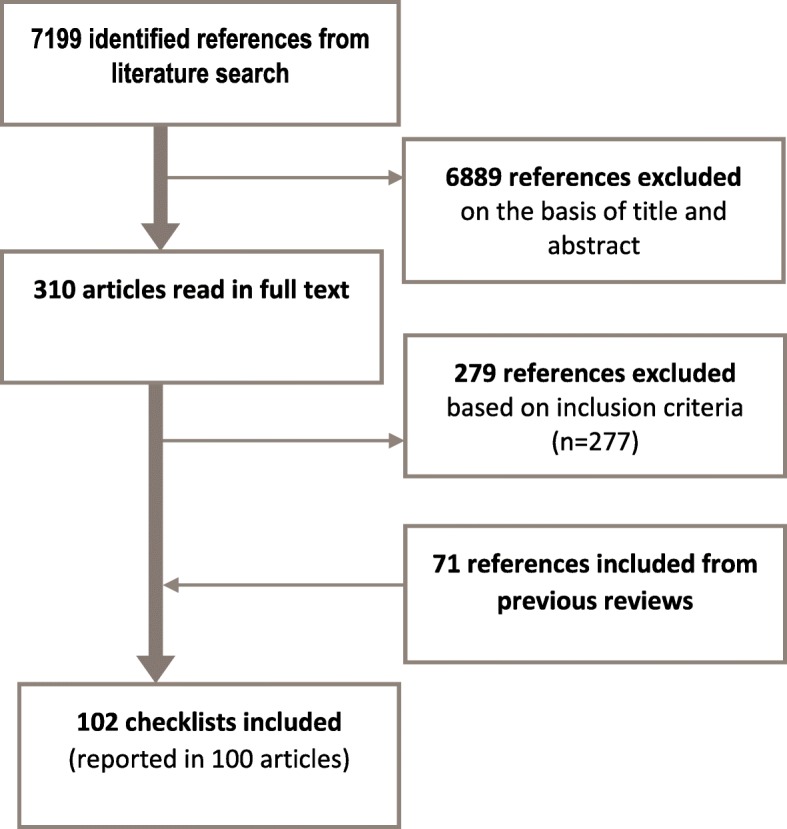
PRISMA Flow chart. Results of systematic mapping review described in this article

This lack of a uniform approach mirrors the situation for systematic reviews of effectiveness over a decade ago, where over 30 checklists were being used to assess the quality of randomised trials [29]. To address this lack of consistency and to reach consensus, a working group of methodologists, editors and review authors developed the risk of bias tool that is now used for Cochrane intervention reviews and is a key component of the GRADE approach [4, 30, 31]. The Cochrane risk of bias tool encourages review authors to be transparent and systematic in how they appraise the methodological limitations of primary studies. Assessments using this tool are based on an assessment of objective goals and on a judgment of whether failure to meet these objective goals raises any concerns for the particular research question or review finding. Similar efforts are needed to develop a critical appraisal tool to assess methodological limitations of primary qualitative studies in the context of qualitative evidence syntheses (Fig. 1).

### Previous reviews

While at least five methodological reviews of critical appraisal tools for qualitative research have been published since 2003, we assessed that these did not adequately address the aims of this project [22–24, 32, 33]. Most of the existing reviews focused only on critical appraisal tools in the health sciences [22–24, 32] . One review focused on reporting standards for qualitative research [23], one review did not use a systematic approach to searching the literature [24], one review included critical appraisal tools for any study design (quantitative or qualitative) [32], and one review only included tools defined as “‘high-utility tools’ […] that are some combination of available, familiar, authoritative and easy to use tools that produce valuable results and offer guidance for their use” [33]. In the one review that most closely resembles the aims of the current review, the search was conducted in 2010, did not include tools used in the social sciences, and was not conducted from the perspective of the GRADE-CERQual approach (see discussion below) [22].

### Current review

We conducted this review of critical appraisal tools for qualitative research within the context of the GRADE-CERQual approach. This reflects our specific interest in identifying (or developing, if need be) a critical appraisal tool to assess the methodological strengths and limitations of a body of evidence that contributes to a review finding and, ultimately, to contribute to an assessment of how much confidence we have in review findings based on these primary studies [3]. Our focus is thus not on assessing the overall *quality* of an individual study, but rather on assessing how any identified methodological limitations of a study could influence our confidence in an individual review finding. This particular perspective may not have exerted a large influence on the conduct of our current mapping review. However, it will likely influence how we interpret our results, reflecting our thinking on methodological limitations both at the individual study level and at the level of a review finding. Our team is also guided by how potential concepts found in existing checklists may overlap with the other components of the GRADE-CERQual approach, namely relevance, adequacy and coherence (see Table 1 for definitions).

**Table 1:** GRADE-CERQual

Component	Definitions
Methodological limitations	The extent to which there are concerns about the design or conduct of the primary studies that contributed evidence to an individual review finding
Coherence	An assessment of how clear and cogent the fit is between the data from the primary studies and a review finding that synthesizes that data. By “cogent” we mean well supported or compelling
Adequacy	An overall determination of the degree of richness and quantity of data supporting a review finding
Relevance	The extent to which the body of evidence from the primary studies supporting a review finding is applicable to the context (perspective or population, phenomenon of interest, setting) specified in the review question

*Reprinted from Lewin and colleagues (2018)* [5]

## Aim

The aim of this review was to systematically map existing critical appraisal tools for primary qualitative studies, and identify common criteria across these tools.

## Methodology

### Eligibility criteria

For the purposes of this review, we defined a critical appraisal tool as a tool, checklist or set of criteria that provides guidance on how to appraise the methodological strengths and limitations of qualitative research. This could include, for instance, instructions for authors of scientific journals; articles aimed at improving qualitative research and targeting authors and peer reviewers; and chapters from qualitative methodology manuals that discuss critical appraisal.

We included critical appraisal tools if they were explicitly intended to be applicable to qualitative research. We included tools that were defined for mixed methods if it was clearly stated that their approach included qualitative methods. We included tools with clear criteria or questions intended to guide the user through an assessment of the study. However, we did not include publications where the author discussed issues related to methodological rigor of qualitative research but did not provide a list or set of questions or criteria to support the end user in assessing the methodological strengths and limitations of qualitative research. These assessments were sometimes challenging, and we have sought to make our judgements as transparent as possible. We did not exclude tools based on how their final critical appraisal assessments were determined (e.g., whether the tool used numeric quality scores, a summary of elements, or weighting of criteria).

We included published or unpublished papers that were available in full text, and that were written in any language, but with an English abstract.

### Search strategy

We began by conducting a broad scoping search of existing reviews of critical appraisal tools for qualitative research in Google Scholar using the terms “critical appraisal OR quality AND qualitative”. We identified four reviews, the most recent of which focussed on checklists used within health sciences and was published in 2016 (search conducted in 2010) [34]. We included critical appraisal tools identified by these four previous reviews if they met the inclusion criteria described above [22–24, 32]. We proceeded to search systematically in health and medical databases for checklists published after 2010 (so as not to duplicate the most recent review described above). Since we were not aware of any review which searched specifically for checklists used in the social sciences, we extended our search in social sciences databases backwards to 2006. We chose this date as our initial reading had suggested that development of critical appraisal within the social science field was insufficiently mature before 2006, and considered that any exceptions would be identified through searching reference lists of identified studies. We also searched references of identified relevant papers and contacted methodological experts to identify any unpublished tools.

In June 2016, we conducted a systematic literature search of Pubmed/MEDLINE, PsycInfo, CINAHL, ERIC, ScienceDirect, Social services abstracts and Web of Science databases using variations of the following search strategy: (“Qualitative research” OR “qualitative health research” OR “qualitative study” OR “qualitative studies” OR “qualitative paper” OR “qualitative papers”) AND (“Quality Assessment” OR “critical appraisal” or “internal validity” or “external validity” OR rigor or rigour) AND (Checklist or checklists or guidelines or criteria or standards) (see Additional file 1 for the complete search strategy). A Google Scholar alert for frequently cited articles and checklists was created to identify any tools published since June 2016.

### Study selection

Using the Covidence web-based tool [35] two authors independently assessed titles and abstracts and then assessed the full text versions of potentially relevant checklists using the inclusion criteria described above. A third author mediated in cases of disagreement.

### Data extraction

We extracted data from every included checklist related to study characteristics (title, author details, year, type of publication), checklist characteristics (intended end user (e.g. practitioner, guideline panel, review author, primary researcher, peer reviewer), discipline (e.g. health sciences, social sciences), and details regarding how the checklist was developed or how specific checklist criteria were justified). We also extracted the checklist criteria intended to be assessed within each identified checklist and any prompts, supporting questions, etc. Each checklist item/question (and supporting question/prompt) was treated as a separate data item. The data extraction form is available in Additional file 2.

### Synthesis methods

We analysed the criteria included in the identified checklists using the best fit framework analysis approach [36]. We developed a framework using the ten items from the Critical Appraisal Skills Programme (CASP) Qualitative Research Checklist. We used this checklist because it is frequently used in qualitative evidence syntheses [28]. We then extracted the criteria from the identified checklists and charted each checklist question or criterion into one of the themes in the framework. We expanded the initial framework to accommodate any coded criteria that did not fit into an existing framework theme. Finally, we tabulated the frequency of each theme across the identified checklists (the number of checklists for which a theme was mentioned as a checklist criterion). The themes, which are derived from the expanded CASP framework, could be viewed as a set of overarching criterion statements based on synthesis of the multiple criteria found in the included tools. However, for simplicity we use the term ‘theme’ to describe each of these analytic groups.

In this paper, we use the terms “checklist” and “critical appraisal tools” interchangeably. The term “guidance” however is defined differently within the context of this review, and is discussed in the discussion section below. The term “checklist criteria” refers to criteria that authors have included in their critical appraisal tools. The term “theme” refers to the 22 framework themes that we have developed in this synthesis and into which the criteria from the individual checklists were sorted. The term “cod(e)/ing” refers to the process of sorting the checklist criteria within the framework themes.

## Results

Our systematic search resulted in 7199 unique references. We read the full papers for 310 of these, and included 31 checklists that met the inclusion criteria. We also included 71 checklists from previous reviews that met our inclusion criteria. A total of 102 checklists were described in 100 documents [22–24, 26, 37–132] (see Fig. 1). A list of the checklists are included in Additional file 3. One publication described three checklists (Silverman 2008; [119]).

### Characteristics of the included checklists

**Fig. 2:**
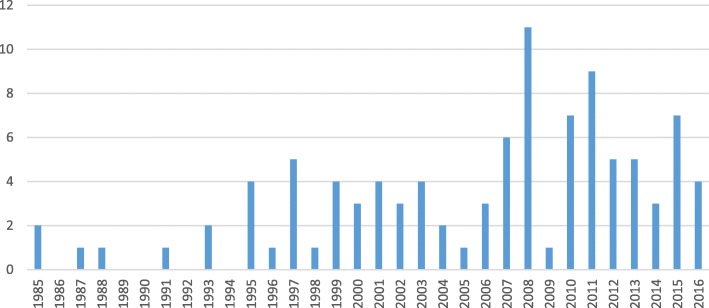
Identified critical appraisal tools (sorted by publication year). References list of critical appraisal tools included in this mapping review

The incidence of new critical appraisal tools appears to be increasing (see Fig. 2). Approximately 80% of the identified tools have been published since 2000.

### Critical appraisal tool development

Approximately half of the articles describing critical appraisal tools did not report how the tools were developed, or this was unclear (*N* = 53). Approximately one third of tools were based on a review and synthesis of existing checklists (*N* = 33), or adapted directly from one or more existing checklists (*N* = 10). The other checklists were developed using a Delphi survey method or consultation with methodologists or practitioners (*N* = 4), a review of criteria used by journal peer reviewers (N = 1), or using a theoretical approach (N = 1).

#### Health or social welfare field

We attempted to sort the checklists according to the source discipline (field) in which they were developed (e.g. health services or social welfare services). In some cases this was apparent from the accompanying article, or from the checklist criteria, but in many cases we based our assessment on the authors’ affiliations and the journal in which the checklist was published. The majority of checklists were developed by researchers in the field of health care (*N* = 60). The remaining checklists appear to have been developed within health and/or social care (*N* = 2), education (N = 2), social care (*N* = 4), or other fields (*N* = 8). Many publications either did not specify any field, or it was unclear within which field the checklist was developed (*N* = 26).

#### Intended end user

It was unclear who the intended end user was (e.g., policy maker, clinician/practitioner, primary researcher, systematic review author, or peer reviewer) for many of the checklists (*N* = 34). Of the checklists where the intended end user was implied or discussed, ten were intended for primary authors and peer reviewers, and ten were intended for peer reviewers alone. Seventeen checklists were intended to support practitioners in reading/assessing the quality of qualitative research, and 17 were intended for use by primary researchers to improve their qualitative research. Ten checklists were intended for use by systematic review authors, two for use by primary research authors and systematic review authors, and two were intended for students appraising qualitative research.

#### Checklist versus guidance

The critical appraisal tools that we identified appeared to vary greatly in how explicit the included criteria were and the extent of accompanying guidance and supporting questions for the end user. Below we discuss the differences between checklists and guidance with examples from the identified tools.

### Checklist

Using the typology described by Hammersley (2007), the term “checklist” is used to describe a tool where the user is provided with observable indicators to establish (along with other criteria) whether or not the findings of a study are valid, or are of value. Such tools tend to be quite explicit and comprehensive; furthermore the checklist criteria are usually related to research conduct and may be intended for people unfamiliar with critically appraising qualitative research [8]. The tool described in Sandelowski (2007) is an example of such a checklist [115].

### Guidance

Other tools may be intended to be used as guidance, with a list of considerations or reminders that are open to revision when being applied [8]. Such tools are less explicit. The tool described by Carter (2007) is such an example, where the focus on a fundamental appraisal of methods and methodology seems directed at experienced researchers [48].

### Results of the framework synthesis

Through our framework synthesis we have categorised the criteria included in the 102 identified critical appraisal tools into 22 themes. The themes represent a best effort at translating many criteria, worded in different ways, into themes. Given the diversity in how critical appraisal tools are organized (e.g. broad versus narrow questions), not all of the themes are mutually exclusive (e.g. some criteria are included in more than one theme if they address two different themes), and some themes are broad and include a wide range of criteria from the included critical appraisal tools (e.g. *Was the data collected in a way that addressed the research issue?* represents any criterion from an included critical appraisal tool that discussed data collection methods). In Table 2, we present the number of criteria from critical appraisal tools that relate to each theme. None of the included tools contributed criteria to all 22 themes.

**Table 2:** Final themes included in the framework

Framework themes^a^	Number of critical appraisal tools that included questions related to theme
Was there a statement of the aims of the research?	59
Did the authors include/discuss a theoretical perspective?	31
Did the authors conduct a review of the literature?	27
Is a qualitative method appropriate?	38
Is this a qualitative study?	4
Was the research design appropriate to address the aims of the research?	62
Were end users involved in the development of the research study?	1
Who are the participants, how were they selected and were the methods for selection appropriate?	75
Was the data collected in a way that addressed the research issue?	79
Did the researcher spend sufficient time in the research setting?	12
Has the research team considered their role in the research process and any influence it may have on the research process or findings?	71
Have ethical issues been taken into consideration?	42
Was the data analysis sufficiently rigorous?	89
Is there a clear statement of findings?	95
How valuable is the research?	71
Have authors discussed/assessed the overall rigor of the research study including strengths and limitations of the research?	31
Is there an audit trail?	22
Did the authors consider/report practicalities of conducting project, and were they realistic?	2
Did the researchers achieve saturation?	11
Was there disclosure of funding sources?	6
Are the authors credible?	8
Reporting criteria (including demographic features of the study)	38

^*a*^*We have attempted to report the framework themes in order of how one would normally read a qualitative research study (*e.g.*, from statement of aims, to clear statement of findings)*

#### Framework themes: design and/or conduct of qualitative research

The majority of the framework themes relate to the design and conduct of a qualitative research study. However, some themes overlap with, or relate to, what are conventionally considered to be reporting standards. The first reporting standards for primary qualitative research were not published until 2007 and many of the appraisal tools predate this and include a mix of methodological quality and quality of reporting standards [23]. The current project did not aim to distinguish or discuss which criteria is related to critical appraisal versus reporting standards. However, we discuss the ramifications of this blurry distinction below.

#### Breadth of framework themes

Some themes represent a wide range of critical appraisal criteria. For example, the theme “Was the data analysis sufficiently rigorous?” includes checklist criteria related to several different aspects of data analysis: (a) whether the researchers provide in-depth description of the analysis process, (b) whether the researchers discuss how data were selected for presentation, (c) if data were presented to support the finding, and (d) whether or not disconfirming cases are discussed. On the other hand, some of the themes cover a narrower breadth of criteria. For example, the theme “Have ethical issues been taken into consideration?” only includes checklist criteria related to whether the researchers have sought ethical approval, informed participants about their rights, or considered the needs of vulnerable participants. The themes differ in terms of breadth mainly because of how the original coding framework was structured. Some of the themes from the original framework were very specific and could be addressed by seeking one or two pieces of information from a qualitative study (e.g., Is this a qualitative study?). Other themes from the original framework were broad and a reader would need to seek multiple pieces of information in order to make a clear assessment (e.g., Was the data collected in a way that addressed the research issue?).

#### Scope of existing critical appraisal tools

We coded many of the checklist criteria as relevant to multiple themes. For example, one checklist criterion was: “Criticality - Does the research process demonstrate evidence of critical appraisal” [128]. We interpreted and coded this criterion as relevant to two themes: “Was the data analysis sufficiently rigorous” and “Is there a clear statement of findings?”. On the other hand, several checklists also contained multiple criteria related to one theme. For instance, one checklist (Waterman 2010; [127]) included two separate questions related to the theme “Was the data collected in a way that addressed the research issue?” (Question 5: Was consideration given to the local context while implementing change? Is it clear which context was selected, and why, for each phase of the project? Was the context appropriate for this type of study? And Question 11: Were data collected in a way that addressed the research issue? Is it clear how data were collected, and why, for each phase of the project? Were data collection and record-keeping systematic? If methods were modified during data collection is an explanation provided?) [127]. A further example relates to reflexivity. The majority of critical appraisal tools include at least one criterion or question related to reflexivity (*N* = 71). Reflexivity was discussed with respect to the researcher’s relationship with participants, their potential influence on data collection methods and the setting, as well as the influence of their epistemological or theoretical perspective on data analysis. We grouped all criteria that discussed reflexivity into one theme.

## Discussion

The growing number of critical appraisal tools for qualitative research reflects increasing recognition of the value and use of qualitative research methods and their value in informing decision making. More checklists have been published in the last six years than in the preceding decade. However, upon closer inspection, many recent checklists are published adaptations of existing checklists, possibly tailored to a specific research question, but without any clear indication of how they improve upon the original. Below we discuss the framework themes developed from this synthesis, specifically which themes are most appropriate for critically appraising qualitative research and why, especially within the context of conducting a qualitative evidence synthesis. We will also discuss differences between checklists and guidance for critical appraisal and the unclear boundaries between critical appraisal criteria and reporting standards.

### Are these the best criteria to be assessing?

The framework themes we present in this paper vary greatly in terms of how well they are covered by existing tools. However, a theme’s frequency is not necessarily indicative of the perceived or real importance of the group of criteria it encapsulates. Some themes appear more frequently than others in existing checklists simply due to the number of checklists which adapt or synthesise one of more existing tools. Some themes, such as “Was there disclosure of funding sources?”, and “Were end users involved in the development of the research study?” were only present in a small number of tools. These themes may be as important as more commonly covered themes when assessing the methodological strengths and limitations of qualitative research. It is unclear whether some of the identified themes were included in many different tools because they actually represent important issues to consider when assessing whether elements of qualitative research design or conduct could weaken our trust in the study findings, or whether frequency of a theme simply reflects a shared familiarity with concepts and assumptions on what constitutes or leads to rigor in qualitative research.

Only four of the identified critical appraisal tools were developed with input from stakeholders using consensus methods, although it is unclear how consensus was reached, or what it was based on. In more than half of the studies there was no discussion of how the tool was developed. *None* of the identified critical appraisal tools appear to be based on empirical evidence or explicit hypotheses regarding the relationships between components of qualitative study design and conduct and the trustworthiness of the study findings. This is directly in contrast to Whiting and colleagues (2017) discussion of how to develop quality assessment tools: “[r]obust tools are usually developed based on empirical evidence refined by expert consensus” [133]. A concerted and collaborative effort is needed in the field to begin thinking about why some criteria are included in critical appraisal tools, what is current knowledge on how the absence of these criteria can weaken the rigour of qualitative research, and whether there are specific approaches that strengthen data collection and analysis processes.

### Methodological limitations: assessing individual studies versus individual findings

**Fig. 3:**
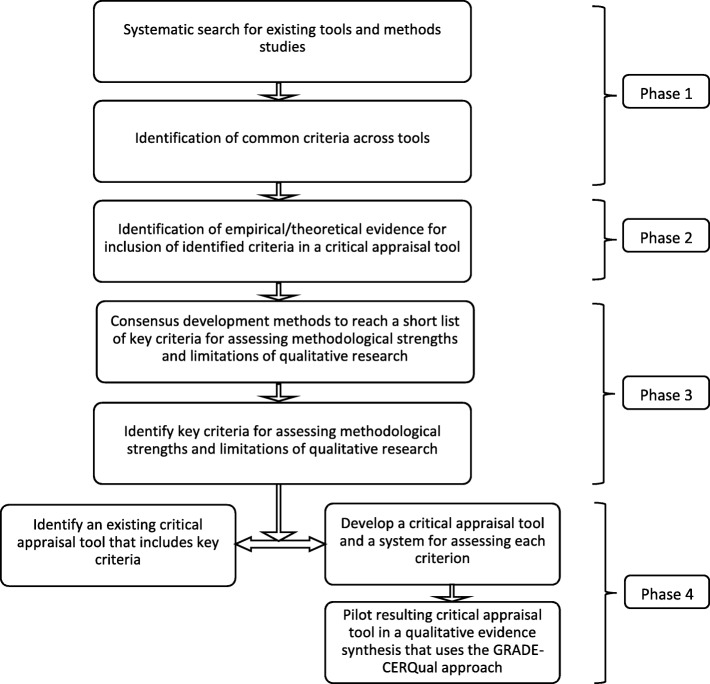
Process of identifying/developing a tool to support assessment of the GRADE-CERQual methodological limitations component (Cochrane qualitative Methodological Limitations Tool; CAMELOT). The research described in this article addresses phase 1 of this project

Thus far, critical appraisal tools have focused on assessing the methodological strengths and limitations of individual studies and the reviews of critical appraisal tools that we identified took the same approach. This mapping review is the first phase of a larger research project to consider how best to assess methodological limitations in the context of qualitative evidence syntheses. In this context, review authors need to assess the methodological “quality” of all studies contributing to a review finding, and also whether specific limitations are of concern for a particular finding as “individual features of study design may have implications for some of those review findings, but not necessarily other review findings” [134]. The ultimate aim of this research project is to identify, or develop if necessary, a critical appraisal tool to systematically and transparently support the assessment of the methodological limitations component of the GRADE-CERQual approach (see Fig. 3), which focuses on how much confidence can be placed in individual qualitative evidence synthesis findings.

### Critical appraisal versus reporting standards

While differences exist between criteria for assessing methodological strengths and limitations and criteria for assessing the reporting of research, the difference between these two aims, and the tools used to assess these, is not always clear. As Moher and colleagues (2014) point out “[t]his distinction is, however, less straightforward for systematic reviews than for assessments of the reporting of an individual study, because the reporting and conduct of systematic reviews are, by nature, closely intertwined” [135]. Review authors are sometimes unable to differentiate poor reporting from poor design or conduct of a study. Although current guidance recommends a focus on criteria related to assessing methodological strengths and limitations when choosing a critical appraisal tool (see discussion in introduction), deciding what is methodological versus a reporting issue is not always straightforward: “without a clear understanding of how a study was done, readers are unable to judge whether the findings are reliable” [135]. The themes identified in the current framework synthesis illustrate this point: while many themes clearly relate to the design and conduct of qualitative research, some themes could also be interpreted as relating to reporting standards (e.g., *Was there disclosure of funding sources? Is there an audit trail).* At least one theme, ‘Reporting standards (including demographic characteristics of the study)’, would not be considered key to assessment of methodological strengths and limitations of qualitative research.

Finally, the unclear distinction between critical appraisal and reporting standards can be demonstrated by the description of one of the tools included in this synthesis [96]. This tool is called Standards for Reporting Qualitative Research (SRQR), however, the tool is both based on a review of critical appraisal criteria from previously published instruments, and concludes that the proposed standards will provide “clear standards for reporting qualitative research” and assist “readers when critically appraising […] study findings” [96] p.1245).

Reporting standards are being developed separately and discussion of these is beyond the remit of this paper [136]. However, when developing critical appraisal tools, one must be aware that some criteria or questions may also relate to reporting and ensure that such criteria are not used to assess both the methodological strengths and limitations *and* reporting quality for a publication.

### Intended audience

This review included any critical appraisal tool intended for application to qualitative research, regardless of the intended end user. The type of end user targeted by a critical appraisal tool could have implications for the tool’s content and form. For instance, tools designed for practitioners who are applying the findings from an individual study to their specific setting may focus on different criteria than tools designed for primary researchers undertaking qualitative research. However, since many of the included critical appraisal tools did not identify the intended end user, it is difficult to establish any clear patterns between the content of the critical appraisal tools and the audience for which the tool was intended. It is also unclear whether or not separate critical appraisal tools are needed for different audiences, or whether one flexible appraisal tool would suffice. Further research and user testing is needed with existing critical appraisal tools, including those under development.

Tools or guidance intended to support primary researchers undertaking qualitative research in establishing rigour were not included in this mapping and analysis. This is because guidance for primary research authors on how to design and conduct high quality qualitative research focus on how to apply methods in the best and most appropriate manner. Critical appraisal tools, however, are instruments used to fairly and rapidly assess methodological strengths and limitations of a study post hoc. For these reasons, those critical appraisal tools we identified and included that appear to target primary researchers as end users may be less relevant than other identified tools for the aims of this project.

### Lessons from the development of quantitative research tools on risk of bias

While the fundamental purposes and principles of qualitative and quantitative research may differ, many principles from development of the Cochrane Risk of Bias tool transfer to developing a tool for the critical appraisal of qualitative research. These principles include avoiding quality scales (e.g. summary scores), focusing on internal validity, considering limitations as they relate to individual results (findings), the need to use judgment in making assessments, choosing domains that combine theoretical and empirical considerations, and a focus on the limitations as represented in the research (as opposed to quality of reporting) [31]. Further development of a tool in the context of qualitative evidence synthesis and GRADE-CERQual needs to take these principles into account, and lessons learned during this process may be valuable for the development of future critical appraisal or Risk of Bias tools.

### Further research

As discussed earlier, CERQual is intended to be applied to individual findings from qualitative evidence syntheses with a view to informing decision making, including in the context of guidelines and health systems guidance [137]. Our framework synthesis has uncovered three important issues to consider when critically appraising qualitative research in order to support an assessment of confidence in review findings from qualitative evidence syntheses. First, since no existing critical appraisal tool describes an empirical basis for including specific criteria, we need to begin to identify and explore the empirical and theoretical evidence for the framework themes developed in this review. Second, we need to consider whether the identified themes are appropriate for critical appraisal within the specific context of the findings of qualitative evidence syntheses. Thirdly, some of the themes from the framework synthesis relate more to research reporting standards than to research conduct. As we plan to focus only on themes related to research conduct, we need to reach consensus on which themes relate to research conduct and which relate to reporting (see Fig. 2).

## Conclusion

Currently, more than 100 critical appraisal tools exist for qualitative research. This reflects an increasing recognition of the value of qualitative research. However, none of the identified critical appraisal tools appear to be based on empirical evidence or clear hypotheses related to how specific elements of qualitative study design or conduct influence the trustworthiness of study findings. Furthermore, the target audience for many of the checklists is unclear (e.g., practitioners or review authors), and many identified tools also include checklist criteria related to reporting quality of primary qualitative research. Existing critical appraisal tools for qualitative studies are thus not fully fit for purpose in supporting the methodological limitations component of the GRADE-CERQual approach. Given the number of tools adapted from previously produced tools, the frequency count for framework concepts in this framework synthesis does not necessarily indicate the perceived or real importance of each concept. More work is needed to prioritise checklist criteria for assessing the methodological strengths and limitations of primary qualitative research, and to explore the theoretical and empirical basis for the inclusion of criteria.

## Additional files

Additional file 1: Search strategy. (DOCX 14 kb)

Additional file 2: Data extraction form. (DOCX 14 kb)

Additional file 3: List of included critical appraisal tools. (DOCX 25 kb)

## Abbreviations

CASP: Critical Appraisal Skills Programme
CINAHL: The Cumulative Index to Nursing and Allied Health Literature database
ERIC: Education Resources Information Center
GRADE: Grading of Recommendations Assessment, Development, and Evaluation
GRADE-CERQual (CERQual): Confidence in the Evidence from Reviews of Qualitative research
PRISMA: Preferred Reporting Items for Systematic Reviews and Meta-Analyses
QIMG: Cochrane Qualitative & Implementation Methods Group
