# Supplementary material for: Systematic mapping of existing tools to appraise methodological strengths and limitations of qualitative research: first stage in the development of the CAMELOT tool
Source: BMC Med Res Methodol. 2019 Jun 4;19:113. doi: 10.1186/s12874-019-0728-6 (PMC6549363; doi:10.1186/s12874-019-0728-6)
Supplement: Supplementary file 1 — Search strategy. (DOCX 14 kb) [file 12874_2019_728_MOESM1_ESM.docx]

# Additional file 1: Search strategy

**Sciencedirect**

Search date: 10.06.2016

Results: 4471 unique references

Search term: Search results: 4,471 results found for (“Qualitative research” OR “qualitative health research” OR “qualitative study” OR “qualitative studies” OR “qualitative paper” OR “qualitative papers”) AND (“Quality Assessment” OR “critical appraisal” or “internal validity” or “external validity” OR rigor or rigour) AND (Checklist or checklists or guidelines or criteria or standards) AND LIMIT-TO(yearnav, "2016,2015,2014,2013,2012,2011,2010").

**PubMed**

Search date: 10.06.2016

Results: 224 unique references

Search term: (“Qualitative research” OR “qualitative health research” OR “qualitative study” OR “qualitative studies” OR “qualitative paper” OR “qualitative papers”) AND (“Quality Assessment” OR “critical appraisal” or “internal validity” or “external validity” OR rigor or rigour OR appraising) AND (Checklist or checklists or guidelines or criteria or standards or tool or tools) TIME LIMIT 2010, 2011, 2012, 2013, 2014, 2015, 2016

**CINAHL**

Search date: 10.06.2016

Results: 84 unique references

Search term: (“Qualitative research” OR “qualitative health research” OR “qualitative study” OR “qualitative studies” OR “qualitative paper” OR “qualitative papers”) AND (“Quality Assessment” OR “critical appraisal” or “internal validity” or “external validity” OR rigor or rigour OR appraising) AND (Checklist or checklists or guidelines or criteria or standards or tool or tools) TIME LIMIT 2010-2016

**Web Of Science**

Search date: 16.06.2016

Results: 518 unique references

((Qualitative research OR qualitative health research OR qualitative study OR qualitative studies OR qualitative paper OR qualitative papers)) AND TOPIC: ((“Quality Assessment” OR “critical appraisal” or “internal validity” or “external validity” OR rigor or rigour OR appraising)) AND TOPIC: ((Checklist or checklists or guidelines or criteria or standards or tool or tools))

Refined by: DOCUMENT TYPES: ( ARTICLE ) AND Databases: ( SCIELO OR RSCI OR WOS OR KJD )

Timespan: 2010-2016.

Search language=Auto

**Social Services Abstracts**

Search date: 16.06.2016

Results: 8 unique references

("Qualitative research" OR "qualitative health research" OR "qualitative study" OR "qualitative studies" OR "qualitative paper" OR "qualitative papers") AND ("Quality Assessment" OR "critical appraisal" OR "internal validity" OR "external validity" OR rigor OR rigour OR appraising) AND (Checklist OR checklists OR guidelines OR criteria OR standards OR tool OR tools)

Date: After 2006

Source type

Conference Papers & Proceedings, Dissertations & Theses, Scholarly Journals

Document type

Journal Article

Language

Afrikaans, Arabic, Bulgarian, Catalan, Chinese, Croatian, Czech, Danish, Dutch, English, Finnish, French, German, Hebrew, Hungarian, Indonesian, Italian, Japanese, Lithuanian, Malay, Norwegian, Polish, Portuguese, Romanian, Russian, Slavic language, Slovak, Slovenian, Spanish, Swedish, Turkish

**PsychInfo**

Search date: 16.06.2016

Results: 1852 unique references

Search strategy: ("Qualitative research" OR "qualitative health research" OR "qualitative study" OR "qualitative studies" OR "qualitative paper" OR "qualitative papers") AND ("Quality Assessment" OR "critical appraisal" OR "internal validity" OR "external validity" OR rigor OR rigour OR appraising) AND (Checklist OR checklists OR guidelines OR criteria OR standards OR tool OR tools)

Other limits: After 2010, journal articles
