# Supplementary material for: Systematic mapping of existing tools to appraise methodological strengths and limitations of qualitative research: first stage in the development of the CAMELOT tool
Source: BMC Med Res Methodol. 2019 Jun 4;19:113. doi: 10.1186/s12874-019-0728-6 (PMC6549363; doi:10.1186/s12874-019-0728-6)
Supplement: Supplementary file 2 — Data extraction form. (DOCX 14 kb) [file 12874_2019_728_MOESM2_ESM.docx]

# Additional file 2: Data extraction form

| **Document first author** |  |
| --- | --- |
| **Year** |  |
| **Type of publication** |  |
| **Discipline of authors of tool** |  |
| **Other field/Name of checklist** |  |
| **Aim of checklist** |  |
| **Intended end user** |  |
| **Other end user - specify** |  |
| **Methods of development (specify)** |  |
| **If tool has steps to appraise, specify** |  |
| **Minimum criteria (Y/N)** |  |
| **Specify minimum criteria** |  |
| **Checklist criteria / questions (specify)** |  |
| **Supporting questions (specify where applicable)** |  |
| **Empirical evidence for criteria? (Y/N)** |  |
| **Empirical evidence - if yes, specify** |  |
| **Scoring system (Y/N)** |  |
| **scoring system Y - specify** |  |
| **Weighting system (Y/N)** |  |
| **Weighting system Y - specify** |  |
| **Which qualitative methods is checklist intended for?** |  |
| **Other notes** |  |
