# Supplementary material for: Systematic mapping of existing tools to appraise methodological strengths and limitations of qualitative research: first stage in the development of the CAMELOT tool
Source: BMC Med Res Methodol. 2019 Jun 4;19:113. doi: 10.1186/s12874-019-0728-6 (PMC6549363; doi:10.1186/s12874-019-0728-6)
Supplement: Supplementary file 3 — List of included critical appraisal tools. (DOCX 25 kb) [file 12874_2019_728_MOESM3_ESM.docx]

# Additional file 3. List of included critical appraisal tools

1. **Methods for the development of NICE public health guidance (third edition): Process and methods**. In*.* UK: National Institute for Health and Care Excellence; 2012.

2. Anderson C: **Presenting and evaluating qualitative research**. *American journal of pharmaceutical education* 2010, **74**(8):141.

3. Baillie L: **Promoting and evaluating scientific rigour in qualitative research**. *Nursing standard (Royal College of Nursing (Great Britain) : 1987)* 2015, **29**(46):36-42.

4. Ballinger C: **Demonstrating rigour and quality?** In: *Qualitative research for allied health professionals: Challenging choices.* edn. Edited by (Eds.) LFCB. Chichester, England: J. Wiley & Sons; 2006: 235-246.

5. Bleijenbergh I, Korzilius H, Verschuren P: **Methodological criteria for the internal validity and utility of practice oriented research**. *Quality & quantity* 2011, **45**(1):145-156.

6. Boeije HR, van Wesel F, Alisic E: **Making a difference: towards a method for weighing the evidence in a qualitative synthesis**. *Journal of evaluation in clinical practice* 2011, **17**(4):657-663.

7. Boulton M, Fitzpatrick R, Swinburn C: **Qualitative research in health care: II. A structured review and evaluation of studies**. *Journal of evaluation in clinical practice* 1996, **2**(3):171-179.

8. Britton N, Jones R, Murphy E, Stacy R: **Qualitative research methods in general practice and primary care**. *Family practice* 1995, **12**(1):104-114.

9. Burns N: **Standards for qualitative research**. *Nursing science quarterly* 1989, **2**(1):44-52.

10. Caldwell K, Henshaw L, Taylor G: **Developing a framework for critiquing health research: An early evaluation**. *Nurse education today* 2011, **31**(8):e1-e7.

11. Campbell R, Pound P, Pope C, Britten N, Pill R, Morgan M, Donovan J: **Evaluating meta-ethnography: A synthesis of qualitative research on lay experiences of diabetes and diabetes care**. *Social Science & Medicine* 2003, **56**(4):671-684.

12. Carter S, Little M: **Justifying knowledge, justifying method, taking action: Epistemologies, methodologies, and methods in qualitative research**. *Qualitative health research* 2007, **17**(10):1316-1328.

13. Cesario S, Morin K, Santa-Donato A: **Evaluating the level of evidence of qualitative research**. *Journal of Obstetric, Gynecologic & Neonatal Nursing* 2002, **31**(6):708-714.

14. Cobb AN, Hagemaster JN: **Ten criteria for evaluating qualitative research proposals**. *Journal of Nursing Education* 1987, **26**(4):138-143.

15. Cohen D, Crabtree BF: **Evaluative criteria for qualitative research in health care: Controversies and recommendations**. *The Annals of Family Medicine* 2008, **6**(4):331-339.

16. Cooney A: **Rigour and grounded theory**. *Nurse researcher* 2011, **18**(4):17-22 16p.

17. Côté L, Turgeon J: **Appraising qualitative research articles in medicine and medical education**. *Medical teacher* 2005, **27**(1):71-75.

18. Creswell JW: **Qualitative Procedures. Research design: qualitative, quantitative, and mixed method approaches (2nd ed.)**. Thousand Oaks, CA: Sage Publications; 2003.

19. **10 questions to help you make sense of qualitative research**

20. Crowe M, Sheppard L: **A general critical appraisal tool: An evaluation of construct validity**. *International journal of nursing studies* 2011, **48**(12):1505-1516.

21. Currie G, McCuaig C, Di Prospero L: **Systematically Reviewing a Journal Manuscript: A Guideline for Health Reviewers**. *Journal of Medical Imaging and Radiation Sciences* 2016, **47**(2):129-138.e123.

22. Curtin M, Fossey E: **Appraising the trustworthiness of qualitative studies: Guidelines for occupational therapists**. *Australian occupational therapy journal* 2007, **54**:88-94.

23. Cyr J: **The pitfalls and promise of focus groups as a data collection method**. *Sociological Methods & Research* 2016, **45**(2):231-259.

24. Dixon-Woods M, Shaw RL, Agarwal S, Smith JA: **The problem of appraising qualitative research**. *Quality and Safety in Health Care* 2004, **13**(3):223-225.

25. El Hussein M, Jakubec SL, Osuji J: **Assessing the FACTS: A Mnemonic for Teaching and Learning the Rapid Assessment of Rigor in Qualitative Research Studies**. *Qualitative Report* 2015, **20**(8):1182-1184.

26. Elder NC, Miller WL: **Reading and evaluating qualitative research studies**. *Journal of Family Practice* 1995, **41**(3):279-285.

27. Elliott R, Fischer CT, Rennie DL: **Evolving guidelines for publication of qualitative research studies in psychology and related fields**. *British Journal of Clinical Psychology* 1999, **38**(3):215-229.

28. Farrell SE, Kuhn GJ, Coates WC, Shayne PH, Fisher J, Maggio LA, Lin M: **Critical appraisal of emergency medicine education research: the best publications of 2013**. *Academic emergency medicine : official journal of the Society for Academic Emergency Medicine* 2014, **21**(11):1274-1283.

29. Fawkes C, Ward E, Carnes D: **What evidence is good evidence? A Masterclass in critical appraisal**. *International Journal of Osteopathic Medicine* 2015, **18**(2):116-129.

30. Forchuk C, Roberts J: **How to critique qualitative research articles**. *Canadian Journal of Nursing Research* 1993, **25**(4):47-56.

31. Forman J, Crewsell J, Damschroder L, Kowalski C, Krein S: **Qualitative research methods: Key features and insights gained from use in infection prevention research**. *American Journal of Infection Control* 2008, **36**(10):764-771.

32. Fossey E, Harvey C, McDermott F, Davidson L: **Understanding and evaluating qualitative research**. *Australian and New Zealand Journal of Psychiatry* 2002, **36**(6):717-732.

33. Fujiura GT: **Perspectives on the publication of qualitative research**. *Intellectual and Developmental Disabilities* 2015, **53**(5):323-328.

34. Greenhalgh T, Taylor R: **How to read a paper: Papers that go beyond numbers (qualitative research)**. *BMJ (Clinical research ed)* 1997, **315**(7110):740-743.

35. Greenhalgh T, Wengraf T: **Collecting stories: is it research? Is it good research? Preliminary guidance based on a Delphi study**. *Medical education* 2008, **42**(3):242-247.

36. Gringeri C, Barusch A, Cambron C: **Examining foundations of qualitative research: A review of social work dissertations, 2008-2010**. *Journal of Social Work Education* 2013, **49**(4):760-773.

37. Hoddinott P, Pill R: **A review of recently published qualitative research in general practice. More methodological questions than answers?** *Family practice* 1997, **14**(4):313-319.

38. Inui T, Frankel R: **Evaluating the quality of qualitative research: A Proposal Pro Tem**. *Journal of General Internal Medicine* 1991, **6**(5):485-486.

39. Jeanfreau SG, Jack L, Jr.: **Appraising qualitative research in health education: guidelines for public health educators**. *Health promotion practice* 2010, **11**(5):612-617.

40. Kitto SC, Chesters J, Grbich C: **Quality in qualitative research: Criteria for authors and assessors in the submission and assessment of qualitative research articles for the Medical Journal of Australia**. *Medical Journal of Australia* 2008, **188**(4):243-246.

41. Kneale J, Santry J: **Critiquing qualitative research**. *Journal of Orthopaedic Nursing* 1999, **3**(1):24-32.

42. Kuper A, Lingard L, Levinson W: **Critically appraising qualitative research**. *BMJ (Clinical research ed)* 2008, **337**:687-692.

43. Lane S, Arnold E: **Qualitative research: A valuable tool for transfusion medicine**. *Transfusion* 2011, **51**(6):1150-1153.

44. Lee E, Mishna F, Brennenstuhl S: **How to critically evaluate case studies in social work**. *Research on Social Work Practice* 2010, **20**(6):682-689.

45. Leininger M: **Evaluation criteria and critique of qualitative research studies**. In: *Critical issues in qualitative research methods.* edn. Edited by (Ed.) JM. Thousand Oaks, CA.: Sage Publications; 1993: 95-115.

46. Leonidaki V: **Critical Appraisal in the Context of Integrations of Qualitative Evidence in Applied Psychology: The Introduction of a New Appraisal Tool for Interview Studies**. *Qualitative Research in Psychology* 2015, **12**(4):435-452.

47. **Critical review form - Qualitative studies (Version 2.0)**

48. Lincoln Y, Guba E: **Establishing trustworthiness**. In: *Naturalistic inquiry.* edn. Edited by (Eds.) YLEG. Newbury Park, CA: Sage Publications; 1985: 289-331.

49. Long A, Godfrey M, Randall T, Brettle A, Grant M: **Developing evidence based social care policy and practic. Part 3: Feasibility of undertaking systematic reviews in social care** In*.*: University of Leeds (Nuffield Institute for Health) and University of Salford (Health Care Practice R&D Unit); 2002.

50. Long AF, Godfrey M: **An evaluation tool to assess the quality of qualitative research studies**. *International Journal of Social Research Methodology* 2004, **7**(2):181-196.

51. Malterud K: **Qualitative research: Standards, challenges, and guidelines**. *Lancet* 2001, **358**(9280):483-488.

52. Manuj I, Pohlen TL: **A reviewer's guide to the grounded theory methodology in logistics and supply chain management research**. *International Journal of Physical Distribution & Logistics Management* 2012, **42**(8-9):784-803.

53. Marshall C, Rossman GB: **Defending the value and logic of qualitative research**. In: *Designing qualitative research.* edn. Newbury Park, CA: Sage Publications; 1989.

54. Mays N, Pope C: **Qualitative research: Rigour and qualitative research**. *BMJ (Clinical research ed)* 1995, **311**(109-112).

55. Mays N, Pope C: **Qualitative research in health care: Assessing quality in qualitative research.** *BMJ (Clinical research ed)* 2000, **320**(50-52).

56. Meyrick J: **What is good qualitative research? A first step towards a comprehensive approach to judging rigour/quality**. *Journal of health psychology* 2006, **11**(5):799-808.

57. Miles MB, Huberman AM: **Drawing and verifying conclusions**. In: *Qualitative data analysis: An expanded sourcebook (2nd ed).* edn. Thousand Oaks, CA: Sage Publications; 1997: 277-280.

58. Morse JM: **A review committee's guide for evaluating qualitative proposals**. *Qualitative health research* 2003, **13**(6):833-851.

59. Nelson A: **Addressing the threat of evidence-based practice to qualitative inquiry through increasing attention to quality: A discussion paper**. *International journal of nursing studies* 2008, **45**:316-322.

60. Norena ALP, Alcaraz-Moreno N, Guillermo Rojas J, Rebolledo Malpica D: **Applicability of the Criteria of Rigor and Ethics in Qualitative Research**. *Aquichan* 2012, **12**(3):263-274.

61. O'Brien BC, Harris IB, Beckman TJ, Reed DA, Cook DA: **Standards for reporting qualitative research: a synthesis of recommendations**. *Academic medicine : journal of the Association of American Medical Colleges* 2014, **89**(9):1245-1251.

62. O'Cathain A, Murphy E, Nicholl J: **The quality of mixed methods studies in health services research**. *Journal of health services research & policy* 2008, **13**(2):92-98.

63. O'HEocha C, Wang X, Conboy K: **The use of focus groups in complex and pressurised IS studies and evaluation using Klein & Myers principles for interpretive research**. *Information Systems Journal* 2012, **22**(3):235-256.

64. Oliver DP: **Rigor in Qualitative Research**. *Research on Aging* 2011, **33**(4):359-360 352p.

65. Pearson A, Jordan Z, Lockwood C, Aromataris E: **Notions of quality and standards for qualitative research reporting**. *International journal of nursing practice* 2015, **21**(5):670-676.

66. Peters S: **Qualitative Research Methods in Mental Health**. *Evidence Based Mental Health* 2010, **13**(2):35-40 36p.

67. **Guidelines for Articles. Canadian Family Physician.**

68. Plochg T, Van Zwieten M (eds.): **Guidelines for quality assurance in health and health care research: Qualitative research**. Amsterdam, NL: Qualitative Research Network AMCUvA; 2002.

69. **Proposal: A mixed methods appraisal tool for systematic mixed studies reviews.**

70. Poortman CL, Schildkamp K: **Alternative quality standards in qualitative research?** *Quality & Quantity: International Journal of Methodology* 2012, **46**(6):1727-1751.

71. Popay J, Williams G: **Qualitative research and evidence-based healthcare**. *Journal of the Royal Society of Medicine* 1998, **91**(35):32-37.

72. Ravenek MJ, Rudman DL: **Bridging Conceptions of Quality in Moments of Qualitative Research**. *International Journal of Qualitative Methods* 2013, **12**:436-456.

73. Rice-Lively ML: **Research proposal evaluation form: Qualitative methodology**. Date accessed: 11 October 2016. https://www.ischool.utexas.edu/~marylynn/qreval.html UT School of Information; 1995.

74. Rocco T: **Criteria for evaluating qualitative studies**. *Human Research Development International* 2010, **13**(4):375-378.

75. Rogers A, Popay J, Williams G, Latham M: **Part II: Setting standards for qualitative research: The development of markers**. In: *Inequalities in health and health promotion: Insights from the qualitative research literature* edn. London: Health Education Authority; 1997: 35-52.

76. Rowan M, Huston P: **Qualitative research articles: Information for authors and peer reviewers**. *Canadian Meidcal Association Journal* 1997, **157**(10):1442-1446.

77. Russell CK, Gregory DM: **Evaluation of qualitative research studies**. *Evidence Based Nursing* 2003, **6**(2):36-40.

78. Ryan F, Coughlan M, Cronin P: **Step-by-step guide to critiquing research. Part 2: Qualitative research**. *British Journal of Nursing* 2007, **16**(12):738-744.

79. Salmon P: **Assessing the quality of qualitative research**. *Patient education and counseling* 2013, **90**(1):1-3.

80. Sandelowski M, Barroso J: **Appraising reports of qualitative studies**. In: *Handbook for synthesizing qualitative research.* edn. New York: Springer; 2007: 75-101.

81. Santiago-Delefosse M, Gavin A, Bruchez C, Roux P, Stephen SL: **Quality of qualitative research in the health sciences: Analysis of the common criteria present in 58 assessment guidelines by expert users**. *Social Science & Medicine* 2016, **148**:142-151 110p.

82. Savall H, Zardet V, Bonnet M, Péron M: **The emergence of implicit criteria actually used by reviewers of qualitative research articles**. *Organizational Research Methods* 2008, **11**(3):510-540.

83. Schou L, Hostrup H, Lyngso EE, Larsen S, Poulsen I: **Validation of a new assessment tool for qualitative research articles**. *Journal of advanced nursing* 2012, **68**(9):2086-2094.

84. Shortell S: **The emergence of qualitative methods in health services research**. *Health services research* 1999, **34**(5 Pt 2):1083-1090.

85. Silverman D, Marvasti A: **Quality in Qualitative Research** (Table 15.1). In: *Doing Qualitative Research: A Comprehensive Guide.* edn. Thousand Oaks, CA: Sage Publications; 2008: 257-276.)

86. Silverman D, Marvasti A: **Quality in Qualitative Research** (Table 15.2). In: *Doing Qualitative Research: A Comprehensive Guide.* edn. Thousand Oaks, CA: Sage Publications; 2008: 257-276.)

87. Silverman D, Marvasti A: **Quality in Qualitative Research** (Table 15.3). In: *Doing Qualitative Research: A Comprehensive Guide.* edn. Thousand Oaks, CA: Sage Publications; 2008: 257-276.)

88. Sirriyeh R, Lawton R, Gardner P, Armitage G: **Reviewing studies with diverse designs: the development and evaluation of a new tool**. *Journal of evaluation in clinical practice* 2012, **18**(4):746-752.

89. Spencer L, Ritchie J, Lewis JR, Dillon L: **Quality in qualitative evaluation: A framework for assessing research evidence.** In*.* London: Government Chief Social Researcher's Office; 2003.

90. Stige B, Malterud K, Midtgarden T: **Toward an agenda for evaluation of qualitative research**. *Qualitative health research* 2009, **19**(10):1504-1516.

91. Stiles W: **Evaluating qualitative research**. *Evidence-based mental health* 1999, **4**(2):99-101.

92. Storberg-Walker J: **Instructor's corner: Tips for publishing and reviewing qualitative studies in applied disciplines**. *Human Resource Development Review* 2012, **11**(2):254-261.

93. Tong A, Sainsbury P, Craig J: **Consolidated criteria for reporting qualitative research (COREQ): A 32-item checklist for interviews and focus groups**. *International Journal for Quality in Health Care* 2007, **19**(6):349-357.

94. Tracy SJ: **Qualitative Quality: Eight "Big-Tent" Criteria for Excellent Qualitative Research**. *Qualitative Inquiry* 2010, **16**(10):837-851.

95. Treloar C, Champness S, Simpson PL, Higginbotham N: **Critical appraisal checklist for qualitative research studies**. *Indian journal of pediatrics* 2000, **67**(5):347-351.

96. Walsh D, Downe S: **Appraising the quality of qualitative research**. *Midwifery* 2006, **22**(2):108-119.

97. Waterman H, Tillen D, Dickson R, De Konig K: **Action research: A systematic review and guidance for assessment**. *Health Technology Assessment* 2001, **5**(23):43-50.

98. Whittemore R, Chase SK, Mandle CL: **Validity in qualitative research**. *Qualitative health research* 2001, **11**(4):522-537.

99. Yardley L: **Dilemmas in qualitative health research**. *Psychology & Health* 2000, **15**(2):215-228.

100. Yarris LM, Juve AM, Coates WC, Fisher J, Heitz C, Shayne P, Farrell SE: **Critical Appraisal of Emergency Medicine Education Research: The Best Publications of 2014**. *Academic emergency medicine : official journal of the Society for Academic Emergency Medicine* 2015, **22**(11):1327-1336.

101. Zingg W, Castro-Sanchez E, Secci FV, Edwards R, Drumright LN, Sevdalis N, Holmes AH: **Innovative tools for quality assessment: integrated quality criteria for review of multiple study designs (ICROMS)**. *Public health* 2016, **133**:19-37.

102. Zitomer MR, Goodwin D: **Gauging the Quality of Qualitative Research in Adapted Physical Activity**. *Adapted Physical Activity Quarterly* 2014, **31**(3):193-218.
